# Ion Channel Regulation in Caveolae and Its Pathological Implications

**DOI:** 10.3390/cells14090631

**Published:** 2025-04-24

**Authors:** Jianyi Huo, Liangzhu Mo, Xiaojing Lv, Yun Du, Huaqian Yang

**Affiliations:** Department of Cardiology, The Fourth Affiliated Hospital, Cyrus Tang Medical Institute, Medical College, Soochow University, Suzhou 215028, China; 20234253018@stu.suda.edu.cn (L.M.); 20234253004@stu.suda.edu.cn (X.L.); 20234053004@stu.suda.edu.cn (Y.D.)

**Keywords:** caveolae, ion channels, caveolin, mechanosensation

## Abstract

Caveolae are distinctive, flask-shaped structures within the cell membrane that play critical roles in cellular signal transduction, ion homeostasis, and mechanosensation. These structures are composed of the caveolin protein family and are enriched in cholesterol and sphingolipids, creating a unique lipid microdomain. Caveolae contribute to the functional regulation of various ion channels through both physical interactions and involvement in complex signaling networks. Ion channels localized within caveolae are involved in critical cellular processes such as the generation and propagation of action potentials, cellular responses to mechanical forces, and regulation of metabolism. Dysregulation of caveolae function has been linked to the development of various diseases, including cardiovascular disorders, neurodegenerative diseases, metabolic syndrome, and cancer. This review summarizes the ion channel function and regulation in caveolae, and their pathological implications, offering new insights into their potential as therapeutic targets for ion channel-related diseases.

## 1. Introduction

### 1.1. Discovery of Caveolae and Characterization of Caveolin Proteins

**Figure 1:**
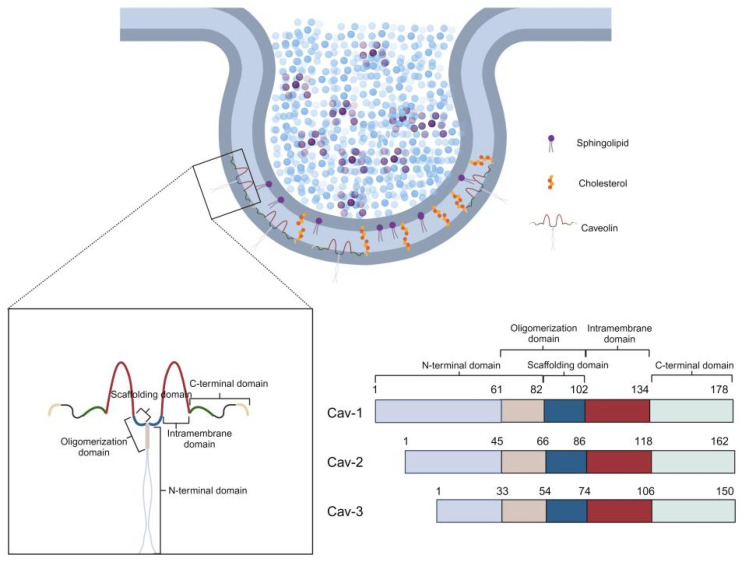
Structure of caveolae and the three caveolin proteins (Cav-1, Cav-2, Cav-3). Caveolin proteins contain distinct domains: an N-terminal domain, a scaffolding domain (SD), an intramembrane domain, and a C-terminal domain.

Caveolae are unique flask-shaped invaginations of the plasma membrane, 50–100 nm in diameter, and were first observed in the mid-20th century thanks to groundbreaking electron microscopy development. In 1953, George E. Palade, during his investigation of endothelial cell ultrastructure, identified these formations as “small pits” or “plasmalemmal vesicles” that appeared to cluster dynamically at the cell surface, indicating their potential involvement in transcellular transport [1]. This groundbreaking finding established the basis for our comprehension of membrane microdomains. Two years afterward, Eichi Yamada officially coined the term caveolae, meaning “little caves within the cell,” in his comprehensive morphological study of gallbladder epithelium, highlighting their widespread occurrence in fibroblasts, adipocytes, and smooth muscle cells [2]. Initial theories viewed caveolae mainly as facilitators of endocytosis. However, subsequent investigation broadened their functional significance to encompass involvement in lipid sorting, mechanotransduction, and cellular signaling [3]. The molecular characteristics of caveolae were not clearly understood until the 1990s, when breakthroughs in protein biochemistry facilitated the identification of caveolin proteins. A pivotal study by Rothberg et al. in 1992 revealed caveolin-1 (Cav-1) as an essential structural element of caveolae. Through experiments with Rous sarcoma virus-transformed fibroblasts, they showed that Cav-1, an integral membrane protein weighing 21–24 kDa, is subject to tyrosine phosphorylation and forms high-molecular-weight oligomers that help maintain the structural integrity of caveolae [4]. Cav-1 has been demonstrated to directly interact with cholesterol through its scaffolding domain, which aids in the curvature of membranes and the formation of caveolae [5]. This finding positioned Cav-1 as a key regulator of caveolar biogenesis and a dependable marker for these microdomains. Further research identified two more mammalian caveolin isoforms. Caveolin-2 (Cav-2), co-discovered by Scherer et al. in 1996, shares about 38% sequence homology with Cav-1 and co-localizes with it in non-muscle tissues, where they form hetero-oligomers crucial for maintaining caveolar stability [6]. Cav-2 does not possess the independent capability to create caveolae and relies on Cav-1 for effective integration into the membrane. On the other hand, caveolin-3 (Cav-3), discovered by Tang et al. in 1996, is a muscle-specific isoform that shares 58% homology with Cav-1 and is essential for caveolae formation in cardiomyocytes, skeletal myocytes, and smooth muscle cells. Importantly, mice lacking Cav-3 show significant muscular dystrophy and impaired sarcolemmal structure, highlighting its critical function in muscle physiology [7]. Up to now, the caveolin protein family in mammals consists of three members: Cav-1, Cav-2, and Cav-3. Among these, only Cav-1 and Cav-3 are directly involved in the formation of caveolae at the plasma membrane [8,9]. Cav-1 is widely present across various cell types, such as endothelial cells, adipocytes, and fibroblasts [4,10,11,12]. Cav-3 expression is primarily limited to striated muscle cells and specific smooth muscle cells [13,14]. Cav-2 does not directly promote the formation of caveolae; however, it associates with the membrane and demonstrates functional activity by hetero-oligomerizing with Cav-1 [6,15]. In cells that do not naturally express Cav-1 or Cav-3, the introduction of either protein significantly enhances the formation of morphologically recognizable caveolae. Conversely, the genetic deletion of these proteins greatly impairs caveolae development [16,17]. Caveolin proteins possess a highly conserved structure characterized by four unique domains (Figure 1): an N-terminal domain, a scaffolding domain, a transmembrane domain, and a C-terminal domain [18,19,20]. They contain a highly conserved signature motif, “FEDVIAEP,” located within their hydrophobic N-terminal region, which is found in multiple species [9,21]. The Cav-1 and Cav-2 genes are situated in proximity on chromosome 7q31.1 of the human genome, with a separation of around 19 kb, while Cav-3 is found on chromosome 3p25. Examination of gene structure and sequence similarity indicates that Cav-2 could be a genomic precursor to both Cav-1 and Cav-3 [22,23]. It has been demonstrated through caveolin knockout mouse models that caveolins play a critical role in various physiological conditions, including muscular dystrophy, cardiomyopathy, obesity, pulmonary hypertension, and tumor development. Notably, the deletion of any individual caveolin gene, or even all caveolin genes, does not lead to lethality [24].

### 1.2. Physiological and Pathological Significance of Ion Channels and Their Association with Caveolae

**Figure 2:**
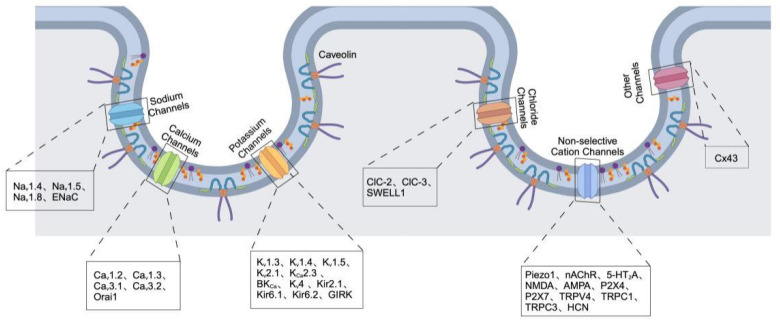
Structure of caveolae and distribution of ion channels. The flask-shaped caveolae structures in the plasma membrane are associated with various ion channels, including sodium channels (Na_v_1.4, Na_v_1.5, Na_v_1.8, ENaC), calcium channels (Ca_v_1.2, Ca_v_1.3, Ca_v_3.1, Ca_v_3.2, Orai1), potassium channels (K_v_1.3, K_v_1.4, K_v_1.5, K_v_2.1, K_Ca_2.3, BK_Ca_, K_v_4, Kir2.1, Kir6.1, Kir6.2, GIRK), chloride channels (CIC-2, CIC-3, SWELL1), non-selective cation channels (Piezo1, nAChR, 5-HT_2_A, NMDA, AMPA, P2X4, P2X7, TRPV4, TRPC1, TRPC3, HCN) and other channels (Cx43).

Ion channels are essential membrane proteins that regulate the movement of ions through cellular membranes, crucial for maintaining membrane potential, facilitating signal transduction, and ensuring cellular homeostasis. Abnormalities in ion channel function are associated with various diseases, such as cardiac arrhythmias, neurodegenerative disorders, and cancer [25]. For instance, voltage-gated sodium channels play a vital role in generating action potentials in excitable cells, whereas calcium-activated potassium channels (K_Ca_) are essential for regulating vascular tone and neuronal excitability [26]. Pathogenic mutations or changes in the expression of these channels interfere with cellular function, underscoring their potential as both therapeutic targets and biomarkers for disease [27]. Caveolae are invaginations in the plasma membrane that are rich in cholesterol and contain caveolin proteins, functioning as active signaling platforms that organize ion channels and modulate their activity. Recent studies indicate that caveolae play a crucial role in the localization, trafficking, and functional regulation of ion channels (Table 1) via interactions between lipids and proteins, as well as through scaffolding proteins [28]. Cav-1 directly engages with L-type calcium channels (Ca_v_1.2) to regulate calcium entry in cardiomyocytes, which is essential for the excitation-contraction coupling process [29]. K_v_1.5 potassium channels are also localized within caveolae in vascular smooth muscle cells, with their function being influenced by the cholesterol levels present in these caveolar structures [30]. Disruption of caveolae architecture, as observed in Cav-1 knockout models, results in abnormal ion channel activity and is associated with cardiovascular diseases and muscular dystrophies [31]. These results highlight the relationship between ion channel function and the integrity of caveolae (Figure 2), offering a mechanistic basis for comprehending the development of diseases.

## 2. Ion Channels Localized in Caveolae

### 2.1. Sodium Channels

#### 2.1.1. Voltage-Gated Sodium Channels (Na_v_)

Voltage-gated sodium channels (Na_v_) are strategically positioned within caveolar microdomains, highlighting their complex spatial organization and specialized functional roles in cellular membranes. Early biochemical studies using sucrose density gradient fractionation have effectively shown that sodium channels are concentrated in caveolin-rich membrane fractions, including areas of the plasma membrane and T-tubule membranes, emphasizing the favored localization of Na_v_ channels within caveolae [32]. The biochemical evidence is complemented by molecular interactions that underscore the strong connection between sodium channels and caveolins. Immunoprecipitation studies have revealed a direct link between Cav-3 and the Na_v_1.4 isoform found in skeletal muscle, indicating a structural relationship that probably enhances specialized signaling and the regulation of channels in muscle cell membranes [33]. Imaging and biochemical studies have demonstrated the colocalization and interaction of Cav-3 with the cardiac-specific sodium channel isoform, Na_v_1.5. This interaction is crucial for regulating electrical excitability and rhythm in cardiomyocytes [34]. Co-immunoprecipitation studies have reinforced this idea by demonstrating the presence of molecular complexes that include Cav-1, Na_v_1.5, and sodium-hydrogen exchanger isoform-1 (NHE-1). This underscores the role of caveolae as active signaling platforms where various membrane proteins come together, potentially facilitating coordinated functional interactions [35,36]. Comprehensive biophysical studies utilizing surface plasmon resonance have validated direct interactions between a key segment (amino acids 85–103) of the voltage-gated sodium channel type-X (Na_v_1.8) α subunit and the scaffolding domain of Cav-1 (amino acids 81–100). This finding sheds light on the molecular mechanism through which Na_v_ channels are selectively recruited and maintained within caveolar membranes [37]. The functional significance of this localization is highlighted by the observed interactions between Cav-3 and Gs_α_ proteins in cardiac myocytes, which occur even without the conventional caveolin-binding motifs. This suggests that sodium channels associated with caveolae may engage in more complex signaling pathways than previously understood, thereby playing a crucial role in regulating cardiac function and cellular excitability [38].

#### 2.1.2. Epithelial Sodium Channels (ENaC)

A key mechanosensitive channel found in caveolae is the epithelial sodium channel (ENaC). These channels are specifically located within caveolar regions on the apical membranes of endothelial cells, where they are closely linked to heme oxygenase-1 (HO-1) and heme oxygenase-2 (HO-2). This arrangement enhances their sensitivity to mechanical forces like stretch and shear stress, indicating that caveolae play a crucial role in the fine-tuned regulation of ENaC-driven sodium transport in reaction to mechanical changes [39]. Biochemical analyses employing detergent-resistant membranes, buoyancy density gradient fractionation, and immunolocalization have confirmed the presence of ENaC in Cav-1-enriched lipid raft compartments within mouse cortical collecting duct cells. This evidence suggests that the localization within caveolar structures could play a crucial role in the stability and functionality of ENaC [40]. Co-immunoprecipitation experiments provide strong evidence for a direct molecular interaction between Cav-1 and ENaC, underscoring the potential regulatory function of Cav-1 in influencing the surface expression and functional behavior of these mechanosensitive sodium channels [41].

### 2.2. Calcium Channels

#### 2.2.1. Voltage-Gated Calcium Channels (Ca_v_)

Calcium channels, especially the voltage-gated L-type (LTCCs), including Ca_v_1.2 and Ca_v_1.3, and T-type (TTCCs), including Ca_v_3.1 and Ca_v_3.2, are uniquely and functionally positioned within caveolar membrane microdomains. This specific localization is crucial for the accurate regulation of calcium signaling, influencing cellular excitability, contractility, and multiple signal transduction pathways. It has been shown through immunofluorescence in neonatal rat cardiomyocytes that overexpressed LTCC β2a subunits significantly colocalize with Cav-3, particularly in their phosphorylated state, highlighting caveolae as specialized sites for channel modulation and signaling interactions [42]. Electron microscopic analyses utilizing immunogold labeling provide compelling evidence for the colocalization of the Ca_v_1.2 with Cav-3 in specific caveolar structures. Notably, distinct β-adrenergic receptor subtypes exhibit selective modulation of different LTCC populations: activation of β_2_-adrenergic receptors specifically increases L-type calcium current through caveolar-localized LTCCs in cardiac cells, whereas β_1_-adrenergic receptors primarily influence LTCCs located outside of caveolae, underscoring a complex and spatially nuanced regulatory mechanism involving caveolae in the heart [43]. Both Ca_v_1.2 and Ca_v_1.3 have been found in caveolar compartments closely associated with Cav-3. However, this interaction shows a decline with age, indicating a dynamic regulation of calcium channel localization throughout the lifespan [44]. Immunofluorescence studies further confirm that Ca_v_1.2 channels are primarily situated within caveolae and other specialized membrane regions, indicating that caveolae could act as essential centers for various signaling interactions related to calcium channels [29]. Recent co-immunoprecipitation research has demonstrated direct interactions between Cav-3 and various calcium channel subtypes, notably Ca_v_1.2 and Ca_v_3.1, highlighting the role of caveolae in structuring specific molecular complexes. These interactions help to compartmentalize essential cardiac pacemaker proteins, including HCN4, Ca_v_1.2, Ca_v_1.3, Ca_v_3.1, and the sodium-calcium exchanger NCX1, into specialized signaling hubs that are vital for maintaining rhythmic electrical activity in cardiac cells [45]. Additional findings from proximity ligation assays conducted on smooth muscle cells from mouse mesenteric arteries validated the specific positioning of the T-type calcium channel Ca_v_3.2 within 40 nm of caveolae marked Cav-1. However, the genetic deletion of Cav-1 disrupts the formation of caveolae and hinders spontaneous transient outward currents, while not directly impacting the activity of the Ca_v_3.2 channel itself [46]. This suggests a complex regulatory mechanism that entails the spatial organization of ion channels without changing their fundamental activity. Immunogold electron microscopy and biochemical assays conducted on ventricular myocytes have demonstrated a distinct colocalization of Ca_v_3.2 channels with Cav-3 within caveolae. This finding is supported by co-immunoprecipitation studies that confirm direct interactions between Ca_v_3.1/Ca_v_3.2 channels and Cav-3. Notably, GST pull-down assays have identified the N-terminal domain of Cav-3 as crucial for its interaction with the Ca_v_3.2 channel, highlighting a specific molecular mechanism for the recruitment of these channels into caveolae and emphasizing the role of caveolae in organizing calcium-dependent signaling pathways [47].

#### 2.2.2. Store-Operated Calcium Channels

Caveolae serve as essential microdomains for store-operated calcium entry (SOCE) channels, notably Orai1. Fluorescence resonance energy transfer analysis, along with immunoprecipitation results, reveals direct molecular interactions between Cav-1 and Orai1, especially during SOCE activation. This interaction plays a crucial role in the dynamic regulation of calcium influx, highlighting the importance of caveolae in maintaining calcium homeostasis [48].

### 2.3. Potassium Channels

#### 2.3.1. Voltage-Gated Potassium Channels (K_v_)

Potassium channels represent a crucial category of voltage-gated ion channels that are strategically positioned within caveolar membrane microdomains, where they are essential for the regulation of cell excitability, metabolism, and signal transduction. Notably, K_v_1-family channels, especially K_v_1.3, are specifically directed to caveolae through direct interactions with Cav-1. During the differentiation of adipocytes, there is a marked increase in K_v_1.3 expression, this protein is targeted to caveolae through its molecular interaction with Cav-1 [49]. This localization holds functional significance, as the integration of K_v_1.3 into caveolar raft structures is essential for the proper functioning of insulin signaling pathways, highlighting the critical role of caveolar organization in metabolic regulation [26].

K_v_1.5 channels exhibit a greater complexity in their association with caveolae. Biochemical analyses and plasma membrane fractionation have confirmed the co-localization of K_v_1.5 with Cav-3 in isolated cardiac caveolar membranes. However, microscopy studies conducted on rat and canine atrial myocytes indicate that there is limited direct spatial overlap between K_v_1.5 and Cav-3, suggesting the possibility of subtle or transient interactions occurring within specific lipid raft subdomains [50,51]. K_v_1.5 are predominantly found in cholesterol-rich membrane microdomains distinct from traditional caveolae. Functional analyses indicate that the depletion of cholesterol leads to a significant increase in channel current density, highlighting the importance of the lipid microenvironment in determining K_v_1.5 functionality [52]. Additionally, the K_v_4 subfamily, which includes K_v_4.1 and K_v_4.3, has been localized within lipid raft domains of pancreatic α-cells, where they are closely associated with Cav-2 and essential signaling and SNARE proteins. This structural configuration suggests that caveolar lipid rafts serve as crucial signaling hubs that regulate hormone secretion and excitability in endocrine cells [53].

K_v_1.4, K_v_1.5, K_v_2.1, K_v_4 or Ca_v_ channels are located on membrane rafts, sometimes in a tissue-specific manner. Specifically, voltage-dependent potassium channels K_v_1.5 and K_v_4.3 are found in caveolae in mouse L-cells and cardiomyocytes, respectively, whereas K_v_2.1 channels reside on non-caveolar membrane rafts in L-cells and neurons [28,54,55,56]. Large-conductance Ca^2+^-activated potassium channels (BK channels), particularly hSlo1c, exhibit functional interactions with Cav-1. Cav-1 not only facilitates intracellular trafficking of hSlo1c to plasma membrane domains but also critically modulates barrier permeability in epithelial cells through their molecular association. This relationship underscores a broader role for Cav-1 as a functional regulator beyond mere structural anchoring, intricately influencing potassium channel-mediated physiological processes [57]. Additionally, the role of Cav-1 as a modulatory scaffold for endothelial BK_Ca_ channels becomes particularly evident under chronic hypoxia conditions, where loss of Cav-1-mediated inhibition enhances BK_Ca_ channel activity, highlighting cholesterol-dependent modulation as a vital aspect of caveolae-mediated control [58].

Small-conductance calcium-activated potassium channels (K_Ca_2.3) are also localized in caveolae. These channels are internalized through a dynamin- and Rab5-dependent process and subsequently recycled via Rab35/EPI64C pathways, which are essential for maintaining their surface expression and functional regulation [59]. The presence of K_Ca_2.3 in caveolar domains is significant for endothelial physiology, particularly as it co-localizes with transient receptor potential channel V4 (TRPV4) in human microvascular endothelial cells, creating a caveolae-dependent signaling complex that does not include intermediate-conductance K_Ca_3.1 channels [60]. This interaction is vital for endothelial function, as the Cav-1 scaffolding of TRPV4/K_Ca_ channels promotes vasodilation in response to shear stress. Additionally, co-immunoprecipitation studies have demonstrated that both TRPV4 and K_Ca_2.3 channels associate with Cav-1 in freshly isolated bovine coronary artery endothelial cells, underscoring the importance of the TRPV4-K_Ca_2.3 complex in caveolae in coronary circulation and shear stress detection [61].

#### 2.3.2. Inwardly Rectifying Potassium Channels

Inwardly rectifying potassium channels, particularly ATP-sensitive potassium (K_ATP_) channels, represent an essential class of ion channels prominently enriched within caveolar microdomains, reflecting their crucial roles in regulating membrane potential, cellular excitability, and metabolic responses. Accumulating studies have elegantly highlighted the functional significance of caveolae in modulating channel localization and activity. For instance, smooth muscle K_ATP_ channels, notably Kir6.1 subunits, have been localized within caveolin-containing membrane fractions, emphasizing caveolae as specialized platforms that coordinate channel distribution and activity [62,63]. Additionally, Kir6.2 channels exhibit clear colocalization with Cav-3 within cardiomyocyte caveolae, especially concentrated at the peripheral regions of isolated cardiac myocytes. This spatial arrangement underscores caveolae as critical sites for organizing K_ATP_ channel function in cardiomyocytes, potentially impacting cardiac electrical stability and myocardial metabolism [64]. Moreover, vascular Kir6.1-containing K_ATP_ channels are selectively targeted to Cav-1-rich membrane fractions within vascular smooth muscle cells under specific physiological conditions, implicating caveolae as central platforms mediating channel responsiveness to metabolic and vasoactive stimuli [65].

Caveolae play a crucial role in the dynamic regulation of Kir2 family channels. Specifically, Kir2.1 channels have been shown to interact with Cav-1, as demonstrated by co-immunoprecipitation studies revealing their direct molecular association. This interaction leads to a functional inhibition of Kir2 channel currents in HEK293 cells when Cav-1 is co-expressed, indicating that Cav-1 serves as a vital negative regulator that modulates Kir2 channel activity through caveolar compartmentalization [66]. Additionally, significant co-localization of Kir6.2 and Cav-3 has been observed in isolated cardiomyocytes, particularly in peripheral regions, underscoring the relevance of this interaction for normal cardiac electrophysiological function [64]. Moreover, G-protein gated inwardly rectifying potassium (GIRK) channels act as essential parasympathetic effectors in cardiac cells, integrating signals from multiple pathways. Following agonist stimulation, GIRK channels transiently co-localize with endothelin receptors within caveolae, but not with bradykinin receptors. This redistribution can be disrupted by methyl-β-cyclodextrin (MβCD) treatment, underscoring the necessity of caveolar integrity for effective receptor-channel coupling [67].

#### 2.3.3. Two-Pore Domain Potassium Channels

Two-pore domain potassium (K_2_P) channels such as TASK-1 and TWIK-1 have been clearly demonstrated to associate with caveolae through interactions with caveolin proteins. Immunofluorescence imaging has elegantly confirmed TASK-1′s prominent colocalization with Cav-3, underscoring caveolae’s role in stabilizing and modulating this potassium channel’s function [68]. Similarly, recent biochemical analyses have identified Cav-1-mediated interactions with both TWIK-1 and TASK channel subtypes, highlighting the strategic localization and functional regulation of these two-pore domain potassium channels within specialized caveolar signaling platforms [69].

### 2.4. Chloride Channels

Chloride channels found in caveolar microdomains are crucial ion channels that play a significant role in regulating cellular volume, mechanical sensitivity, and epithelial barrier function through their specific membrane localization. The chloride channel ClC-2 interacts directly with Cav-1 and the small GTPase Rab5, which aids in the transport of the tight junction protein occludin to apical membranes, thus influencing epithelial integrity and permeability [70]. Additionally, caveolae membranes act as specialized platforms for mechanosensitive chloride channels (MCCs), including ClC-2, ClC-3, and SWELL1 (LRRC8A). Fluorescence resonance energy transfer imaging has confirmed the strong localization of these MCC within caveolae-rich regions, emphasizing the role of caveolae in regulating mechanosensitive chloride signaling [71]. Moreover, the activation of swelling-induced chloride currents from caveolar-localized ClC-3 and SWELL1 channels highlights their functional importance in mechanically responsive caveolar microdomains. These channels are not merely passive components; they form complex interactions with Cav-3, facilitating precise mechanotransduction and rapid cellular responses to mechanical stimuli, thereby underscoring the dynamic role of caveolae in orchestrating mechanosensitive chloride signaling essential for cellular homeostasis [72].

### 2.5. Non-Selective Cation Channels

#### 2.5.1. Mechanosensitive Ion Channels and Ligand-Gated Ion Channels

Mechanosensitive ion channels are a distinct group of ion channels that are intricately located within caveolar microdomains, emphasizing the essential function of caveolae in converting mechanical stimuli into biological reactions. Notably, Piezo1 channels demonstrate significant functional modulation and a dynamic relationship with caveolae. In hypertrophic cardiomyocytes exposed to abdominal aortic constriction and angiotensin II, a reduction in the colocalization of Piezo1 with Cav-3 is associated with increased Piezo1 activity and higher channel expression. Additionally, the experimental disruption of caveolae using MβCD similarly enhances Piezo1 functionality, indicating that caveolae act as negative regulators of Piezo1 activity under normal conditions [73]. Moreover, ligand-gated ion channels, which are crucial for regulating cellular excitability and signal transduction, also show specialized localization within caveolar microdomains, indicating their functional integration into discrete signaling complexes. A key example is the nicotinic acetylcholine receptor (nAChR), which closely associates with Cav-3 at the neuromuscular junction. Cav-3 not only colocalizes with nAChRs but also plays a vital role in their clustering and stability. Cav-3 acts as a new binding partner for muscle-specific kinase (MuSK), which is essential for agrin-induced MuSK phosphorylation and subsequent Rac-1 activation, both of which are critical for the proper aggregation and stabilization of nAChR clusters on muscle membranes. The absence of Cav-3 significantly disrupts nAChR aggregation, underscoring its important structural and regulatory function within caveolae in muscle cells [74].

Additionally, serotonergic receptors, especially the 5-HT receptor subtypes, have been identified as important ligand-gated channels associated with caveolae. The 5-HT_2_A receptor, in particular, engages with Cav-3 when stimulated by serotonin (5-HT), promoting its movement to caveolar membrane regions. This spatial adjustment significantly affects downstream signaling pathways, altering receptor trafficking and expression. Notably, the absence of Cav-3 disrupts the distribution and functional response of 5-HT_2_A receptors, underscoring the role of caveolae in regulating receptor activity, trafficking, and serotonin-driven hypertrophic signaling in cardiomyocytes [75]. Furthermore, caveolae contain crucial neurotransmitter receptors, such as NMDA, AMPA, and various 5-HT subtypes. The presence of NMDA receptors in caveolae allows for interactions with key regulatory molecules like glycine, glutamate, and aspartate, enabling precise modulation of these receptors. The caveolar localization of these receptors facilitates complex downstream signaling cascades initiated by 5-HT stimulation, which may lead to the activation of adenylyl cyclase (AC), which converts adenosine triphosphate (ATP) into 3′,5′-cyclic adenosine monophosphate (cAMP). This, in turn, may activate cAMP-dependent protein kinase and subsequently AMP-activated protein kinase (AMPK), thereby modulating autophagy/mitophagy, linking caveolar positioning to essential metabolic and cellular homeostatic mechanisms [76,77]. Additionally, ATP-gated ion channels, such as purinergic P2X receptors, are also found partially within caveolae. Studies using immunofluorescence and GST pull-down assays have demonstrated that Cav-1 interacts with both P2X4 and P2X7 receptors, placing these ATP-gated channels in specific caveolar membrane compartments. This partial localization in caveolae influences ATP sensitivity and signaling specificity, indicating that caveolae-mediated compartmentalization is vital for modulating purinergic receptor function and responsiveness [78].

#### 2.5.2. Transient Receptor Potential Channels

Transient receptor potential (TRP) channels are precisely localized within caveolar microdomains, indicating a specialized membrane structure that significantly influences their functional characteristics. Notably, TRPV4 channels are closely associated with caveolae, interacting with Cav-1 in the plasma membrane. At physiological temperature, TRPV4 shows strong colocalization with Cav-1, however, treatments that disrupt cholesterol integrity, such as MβCD or pravastatin, lead to altered membrane distribution and decreased colocalization, highlighting the reliance of caveolae on cholesterol for sustaining TRPV4 localization and signaling [79]. Freshly isolated bovine coronary endothelial cells also demonstrate targeted localization of TRPV4 channels within caveolae, alongside K_Ca_2.3 channels, suggesting a functional interplay enabled by caveolar compartmentalization [60,61]. Interestingly, some studies indicate a more complex scenario: cholesterol depletion via MβCD paradoxically increases the membrane expression of TRPV4, Cav-1, and flotillin, yet shows limited direct colocalization, suggesting variations in caveolar association across different cell types [80]. Furthermore, advanced proximity ligation assays have confirmed direct spatial relationships among TRPV4, Cav-1, and BK channels in arterial endothelial cells, underscoring caveolae as central regulatory platforms that integrate these channels for coordinated vascular responses [81]. Additionally, canonical TRP channels like TRPC1 and TRPC3 are also enriched in caveolae, with TRPC1 showing close colocalization and physical interaction with Cav-3 in muscle tissues, further emphasizing the importance of caveolar domains in organizing TRP channel function [82]. Immunofluorescence investigations in arterial smooth muscle have shown a partial colocalization of TRPC1 with Cav-1, which supports the idea that caveolar microdomains act as structural modulators of TRPC-mediated signaling [83]. Additionally, co-immunoprecipitation analyses from cerebral artery lysates indicate direct interactions between Cav-1, TRPC3, and IP_3_ receptor type 1, further emphasizing the role of caveolae as essential signaling hubs for TRPC channel complexes [84].

#### 2.5.3. Hyperpolarization-Activated Cyclic Nucleotide-Gated (HCN) Channels

Hyperpolarization-activated cyclic nucleotide-gated (HCN) channels play a crucial role in regulating cardiac pacemaker function and are specifically localized within caveolae. It has been demonstrated that direct interactions exist between HCN channels and Cav-3 in cardiac cells, indicating that caveolae are essential for maintaining channel stability, surface expression, and overall functional activity. The functional and expression levels of HCN channels are indeed modulated through interactions with Cav-3, thereby contributing to the integrity of cardiac rhythmicity and conduction systems [45,85].

### 2.6. Other Channels

#### Gap Junction Channels

Gap junction channels, primarily composed of connexins like connexin 43 (Cx43), are notably localized within caveolar microdomains, underscoring the active role of caveolae in facilitating intercellular communication rather than serving solely as structural supports. Among the 20 rodent and 21 human connexin isoforms, Cx43 is the most abundantly expressed connexin protein in the heart. As a typical integral membrane protein, Cx43 is synthesized in the rough endoplasmic reticulum, then translocates to the Golgi apparatus where it undergoes folding and oligomerization into connexons. These connexons, referred to as hemichannels (HCs), are then transported along the secretory pathway to their final destination in the plasma membrane [86,87,88,89,90,91,92]. Cardiac Cx43 creates GJCs at intercellular contacts and hemi-channels (HCs) at the peri-junctional plasma membrane and sarcolemmal caveolae/rafts compartments. These GJCs are fundamental for the direct cardiac cell-to-cell transmission of electrical and molecular signals, ensuring synchronous myocardial contraction. Electron microscopic images of adult rat heart cardiomyocytes show Cx43 structures, including the Golgi compartment where Cx43 is oligomerized into HCs, and post-Golgi HCs that are targeted to caveolae/rafts and gap junctions, forming paired HCs that establish functional GJCs between neighboring cardiomyocytes [93]. Indeed, Cx43 shows robust colocalization, co-fractionation, and co-immunoprecipitation with Cav-1. Notably, molecular interactions have identified two distinct Cav-1 domains, the scaffolding domain (residues 82–101) and the C-terminal domain (135–178), as critical sites mediating direct interactions with Cx43. These interactions suggest that caveolae create a complex spatial environment that enhances the organization, stability, and functionality of Cx43 within gap junctions [94]. The compartmentalization of gap junctions mediated by caveolae holds significant relevance across various cell types. In rat epidermal keratinocytes, Cx43 co-immunoprecipitates and colocalizes with Cav-1 and Cav-2, confirming their close spatial relationship. Importantly, this colocalization has also been observed in human epidermal keratinocytes, indicating that caveolar microdomains serve as a reliable structural framework for Cx43-mediated cell communication within the epidermal barrier [95]. Intriguingly, gap junction channels display a dynamic distribution in response to physiological stimuli, such as ATP, which prompts the rapid movement of Cx43 into caveolae, correlating with changes in gap junction communication [96]. Yeast two-hybrid studies further validate the direct interactions between caveolin and connexins, highlighting the active regulatory role of caveolae beyond mere structural support [97].

**Table 1:** Ion channels in caveolae: localization and caveolin interactions.

Channel Type	Localization in Caveolae/Interaction with Caveolins	Caveolin Isoforms Involved	References
**Sodium Channels**			
Na_v_1.4	Localized within muscle caveolae, direct interaction	Cav-3	[32,33]
Na_v_1.5	Caveolae-localized; forms signaling complex affecting cardiac excitability	Cav-1, Cav-3	[34,35,36]
Na_v_1.8	Stabilized within caveolae by direct interaction	Cav-1	[37]
ENaC	Caveolae-localized in endothelial cells; mechanosensitive sodium transport	Cav-1	[39,40,41]
**Calcium Channels**			
Ca_v_1.2	Caveolae-localized; regulate cardiac calcium signaling and pacemaker activity	Cav-3	[29,42,43,44,45]
Ca_v_1.3			
Ca_v_3.1, Ca_v_3.2	Localized in caveolae; regulate cardiac pacing and calcium signaling	Cav-1, Cav-3	[45,46,47]
Orai1	Caveolae localization; modulates calcium influx	Cav-1	[48]
**Potassium Channels**			
K_v_1.3	Localized in caveolar lipid rafts; insulin signaling	Cav-1	[49,98]
K_v_1.4	Tissue-specific localization in caveolar/non-caveolar rafts	Cav-2, Cav-3	[50,51,52,53,54]
K_v_1.5			
K_v_2.1			
K_v_4			
K_Ca_2.3 (SK3)	Localized in caveolae	Cav-1	[59,60,61]
BK Channels (hSlo1c, BK_Ca_)	Localized in caveolae; regulate permeability and hypoxic response	Cav-1	[57,58]
Kir2.1	Localized in caveolae; regulate membrane potential and metabolism	Cav-1, Cav-3	[62,63,64,65,66].
Kir6.1			
Kir6.2			
GIRK	Localized in caveolae	Cav-3	[67]
TASK-1 and TWIK-1	Colocalized with Cav-1/3	Cav-1, Cav-3	[68,69]
**Chloride Channels**			
ClC-2	Localized in caveolae; regulate cell volume and mechanosensitivity	Cav-1, Cav-3	[70,71,72]
ClC-3			
SWELL1			
**Non-selective Cation Channels**			
Piezo1	Colocalized in cardiomyocytes with Cav-3	Cav-3	[73]
nAChR, 5-HT_2_A, NMDA, AMPA, P2X4, P2X7	Localized in caveolae; modulate neurotransmitter responses and excitability	Cav-1, Cav-3	[75,76,77,78,99]
TRPV4, TRPC1, TRPC3	Localized in caveolae; regulate thermal and mechanical responses	Cav-1, Cav-3	[60,61,79,80,81,82,83,84]
HCN	Caveolae-localized; regulate cardiac pacing and rhythmicity	Cav-3	[45,85]
**Other Channels**			
Cx43	Caveolae-localized; regulate intercellular communication and stability	Cav-1, Cav-2	[94,95,96,97]

## 3. Regulation of Ion Channels by Caveolae

### 3.1. Direct Interaction Between Caveolin Scaffolding Domains and Ion Channels

Caveolins, as the structural proteins of caveolae, play a crucial role in modulating ion channel function by directly interacting with specific channel domains. The caveolin scaffolding domain (CSD) acts as a primary mediator in these interactions, recognizing and binding to ion channel proteins through a conserved caveolin-binding motif (CBM), characterized by an arrangement of aromatic amino acids in the sequence QxQxxxxQ (Table 2), where Q represents tyrosine (Y), tryptophan (W), or phenylalanine (F) [100]. These interactions impact channel localization, function, and regulatory mechanisms, influencing physiological and pathological processes. In Kir channels, both the scaffolding and membrane domains of Cav-3 are necessary for proper channel localization within caveolar microdomains, since disruption of either the Kir2.x CBM sequence or the Cav-3 domain alters channel distribution and function [101,102]. Similarly, K_ATP_ channels, specifically the Kir6.2/SUR2A complex, are negatively regulated by Cav-3, with evidence showing that a Cav-3-derived scaffolding domain peptide significantly inhibits channel function [103]. TRP channels are another class of ion channels regulated by caveolin scaffolding domains. The primary Cav-1 binding region in TRPC5 has been mapped to residues 295–322, highlighting a direct molecular interaction [104]. Furthermore, Cav-1 directly associates with TRPC1, particularly its C-terminal domain, playing an essential role in store-operated calcium entry in endothelial cells [105]. Modulation of this interaction, either by increasing Cav-1 scaffolding domain expression or inhibiting calpain activity, has been shown to significantly alleviate lipopolysaccharide -induced cardiac dysfunction and myocyte apoptosis/autophagy [106].

The large-conductance, voltage- and calcium-activated potassium (BK, MaxiK) channel also interacts with Cav-1, with its α-subunit (Slo1) containing a specific sequence (YNMLCFGIY) that facilitates this interaction [107]. This binding appears to serve dual functions: (i) maintaining BK channels in intracellular compartments, thereby limiting surface expression, and (ii) anchoring plasma membrane-resident channels to Cav-1-enriched domains. Importantly, Cav-1 exerts an inhibitory effect on BK channel activity through its scaffolding domain (residues 82–101), a regulation that may be relieved under conditions such as chronic hypoxia, likely via cholesterol-dependent mechanisms [58,108]. In addition, Cx43 has been shown to interact with two distinct regions of Cav-1, the scaffolding domain and the C-terminal domain, suggesting that Cav-1 plays a complex role in regulating intercellular communication through direct channel binding [94].

### 3.2. Lipid-Dependent Modulation of Ion Channel Function

Caveolae possess a unique lipid composition, enriched with cholesterol, sphingolipids (sphingomyelin and glycosphingolipids), and phosphatidylinositol, which are essential for their structural integrity and functional properties [109]. These lipid components play a crucial role in the regulation of ion channels, influencing their localization, stability, and activity within the membrane microdomains.

Cholesterol is a key regulator of ion channel function, exerting both positive and negative effects on different channels through multiple mechanisms. It can directly interact with specific amino acid residues in ion channels or regulate their recruitment into specialized membrane domains such as lipid rafts and caveolae [110]. For example, TRPV4 is localized within cholesterol-rich caveolar domains, and its activity is strongly influenced by its surrounding membrane lipids, including cholesterol and phosphatidylinositol. The depletion of cholesterol has been shown to modulate TRPV4 activation, enhancing calcium influx in a dose-dependent manner [111]. Similarly, cholesterol depletion has profound effects on voltage-gated potassium channels. The interaction of K_V_7.1 and Cav-1 is significantly disrupted upon cholesterol depletion, leading to increased membrane localization of K_v_7.4, mimicking the effect of ciliobrevin D treatment [112]. K_v_1.5, although not localized to caveolae, resides in cholesterol-rich lipid rafts, and its redistribution following cholesterol depletion increases its current-carrying capacity [52]. K_v_2.1 activation at more negative potentials is induced by sphingomyelinase D treatment, highlighting the impact of lipid modifications on channel gating [113]. Cholesterol plays a crucial role in modulating calcium channels in a highly channel-specific manner. For example, cholesterol depletion enhances Ca_V_2.1channel current [114]. In cardiac myocytes, cholesterol depletion affects Orai1 channels by promoting their internalization, leading to reduced calcium influx and altered diffusion of the channel within the cell membrane. Notably, Cav-1 overexpression restores Orai1 localization and function, highlighting the role of cholesterol-rich domains in maintaining calcium channel activity [48].

Beyond cholesterol, polyunsaturated fatty acids (PUFAs), another key component of caveolae, significantly alter membrane microdomain composition and ion channel activity. In cardiac cells, PUFAs modulate sodium channel gating, reduce sodium current, and shorten the action potential duration [115]. They also directly regulate L-type calcium current, reducing calcium transients and calcium-activated membrane currents [116]. Likewise, phosphatidylinositol 4,5-bisphosphate (PIP_2_), a negatively charged phospholipid enriched in caveolae, plays a crucial role in ion channel modulation, particularly affecting Na/Ca exchangers and K_ATP_ channels, both of which are present in cardiac caveolae. Generally, increased PIP_2_ levels activate Kir channels, whereas depletion inhibits their activity, demonstrating the critical role of membrane lipids in potassium channel regulation [117]. The functional importance of lipid-caveolae interactions is further illustrated in vascular regulation.

**Table 2:** Interaction of caveolin proteins/caveolae with lipids and proteins in ion channel regulation.

Caveolin/Caveolae Component	Interaction Partner	Key Interaction Domain or Mechanism	Effect on Ion Channel Localization, Trafficking, or Function	Reference
Caveolin-3	Kir2.x (Kir channels)	Caveolin scaffolding domain (CSD); Caveolin-binding motif (CBM)	Regulates proper localization in caveolae; disruption alters channel distribution and functionand function	[100,101,102]
Caveolin-3	Kir6.2/SUR2A (K_ATP_ channels)	Caveolin scaffolding domain peptide	Negative regulation; inhibits channel function	[103]
Caveolin-1	TRPC5	Amino acids 295–322	Direct binding modulates channel localization and function	[104]
Caveolin-1	TRPC1	C-terminal domain	Regulates store-operated calcium entry; affects cardiac cell survival and function	[105,106]
Caveolin-1	BK (MaxiK) channel (Slo1 <-subunit)	Slo1 sequence: YNMLCFGIY; Caveolin scaffolding domain residues 82–101	Dual function: Limits surface expression; anchors channels in caveolin-rich domains; negatively regulates channel activity	[58,107,108]
Caveolin-1	Connexin 43 (Cx43)	Scaffolding and C-terminal domains	Complex regulation of intercellular communication	[94]
Cholesterol (lipid)	TRPV4	Cholesterol-rich caveolar domains	Cholesterol depletion enhances TRPV4-mediated Ca^2+^ influx	[110,111]
Cholesterol	K_V_7.1/K_V_7.4	Cholesterol-dependent caveolin interactions	Cholesterol depletion disrupts interaction, alters channel localization	[112]
Cholesterol	K_V_1.5	Cholesterol-rich lipid raft localization	Cholesterol depletion increases channel current capacity	[52]
Cholesterol/Sphingolipids	K_V_2.1	Membrane lipid modification by sphingomyelinase D	Induces activation at more negative potentials	[113]
Cholesterol	Ca_V_2.1	Cholesterol depletion	Enhances channel current	[114]
Cholesterol	Orai1	Cholesterol-rich domains	Depletion causes internalization and reduced Ca^2+^ influx; caveolin-1 reverses effects	[48]
PUFAs	Cardiac Na^+^ and L-type Ca^2+^ channels	Lipid microdomain modulation	Reduces currents; shortens action potential duration	[115,116]
PIP_2_	Kir channels; Na^+^/Ca^2+^ exchangers; K_ATP_ channels	Membrane lipid modulation	Increased PIP_2_ activates Kir channels; depletion inhibits activity	[117]

### 3.3. Compartmentalization of Signaling Cascades

**Figure 3:**
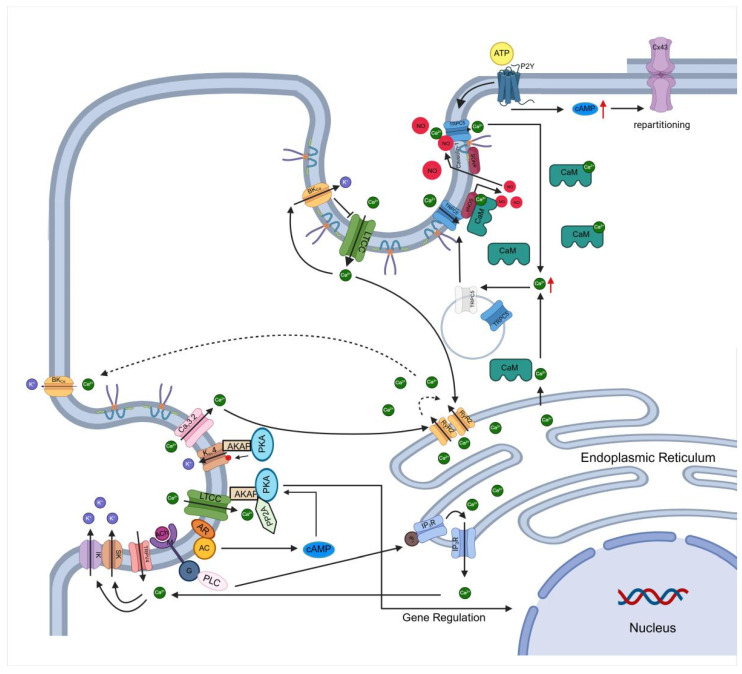
Regulation of ion channels by caveolae and caveolins via signaling pathways. Key signaling components enriched in caveolae include G protein-coupled receptors (GPCRs), heterotrimeric G proteins, steroid hormone receptors, receptor and non-receptor tyrosine kinases, protein kinase A (PKA), protein kinase C (PKC), phosphoinositide 3-kinase (PI3K)/Akt, mitogen-activated protein kinases (MAPK), nitric oxide synthases (NOS) and others.

Caveolae serve as specialized membrane microdomains that compartmentalize key signaling molecules, facilitating efficient and localized signal transduction (Figure 3). These domains are enriched with G protein-coupled receptors (GPCRs), heterotrimeric G proteins, steroid hormone receptors, receptor and non-receptor tyrosine kinases, protein kinase A (PKA) and C (PKC), phosphoinositide 3-kinase (PI3K)/Akt, mitogen-activated protein kinases (MAPK), and nitric oxide synthases (NOS), all of which have been shown to interact directly with caveolins [118]. This spatial organization enables precise regulation of ion channel function through localized cAMP/PKA signaling, nitric oxide (NO)-mediated modulation, and kinase-dependent phosphorylation events.

Caveolae play a critical role in modulating ion channel function through NOS-related signaling cascades. In cardiomyocytes, Cav-3 acts as a negative regulator of late sodium current via nNOS-dependent S-nitrosylation of SCN5A, underscoring the role of caveolae in regulating cardiac excitability [119]. Similarly, TRPC5 activation upon PLC-mediated ATP stimulation triggers Ca^2+^ influx, leading to endothelial NO synthase (eNOS) activation and NO production, which in turn induces further TRPC5 activation via cysteine S-nitrosylation. Mutations in the Cav-1 binding domain of TRPC5 disrupt this interaction, impairing both Ca^2+^ influx and NO production, suggesting that Cav-1 scaffolds TRPC5 and eNOS into a functional signaling complex [104].

Caveolae also serve as key regulators of cAMP/PKA-dependent ion channel modulation by compartmentalizing signaling components. Disrupting caveolae with MβCD reduces whole-cell K_ATP_ currents sensitive to PKA, indicating that caveolar integrity is crucial for adenylyl cyclase (AC)-mediated channel regulation [120]. In ventricular myocytes, α_1_-adrenergic receptor (α_1_-AR) activation modulates transient outward K^+^ current through a PKA-dependent pathway, but this effect remains spatially restricted within caveolae. Components of this pathway, including K_v_4.2/K_v_4.3 channels, PKA, and AKAP100, are assembled in caveolae through interactions with Cav-3, forming a functional microdomain distinct from α_1_-AR, G_α_s, and adenylyl cyclase complexes [54,121]. L-type calcium channels are also targeted to caveolae, where CaMKII-mediated phosphorylation promotes cardiac hypertrophy. Disrupting caveolar integrity with MβCD significantly attenuates this effect, likely due to the loss of PKA-mediated channel phosphorylation and microdomain-specific signaling [42,45].

β-adrenergic receptor (β-AR) signaling is another pathway that is compartmentalized within caveolae. β_2_-ARs are primarily localized to caveolae in the basal state but translocate out of caveolae upon activation, leading to cAMP production by adenylyl cyclase V/VI, which is also highly concentrated within caveolae [122]. Cholesterol depletion disrupts β_2_-AR signaling, suggesting that caveolae act as negative regulators of cAMP accumulation, restricting its diffusion and ensuring localized modulation of ion channels. In ventricular myocytes, β_2_-AR activation leads to localized cAMP production near LTCCs, enhancing Ca^2+^ influx via PKA-dependent phosphorylation without broadly affecting other cAMP-dependent processes [43,44]. This compartmentalization is essential for precise regulation of cardiac pacemaking, as β_2_-ARs in caveolae localize near HCN4, Ca_V_1.2, and Ca_V_1.3 channels, ensuring their coordinated regulation [44].

Caveolae also mediate Angiotensin II (Ang II)-dependent inhibition of K_ATP_ channels in vascular smooth muscle cells. Upon Ang II stimulation, PKCη is selectively recruited to caveolae, where it phosphorylates and inhibits K_ATP_ channels, highlighting the role of caveolae in spatially organizing PKC isoforms for targeted channel modulation [123]. Additionally, caveolae coordinate Ca^2+^-dependent signaling cascades by scaffolding key proteins such as TRPV4, BK_Ca_, and RyRs, which regulate vascular tone and excitation-contraction coupling. In aged VSMCs, the loss of caveolar Ca_V_3.2-RyR coupling alters Ca^2+^ signaling, suggesting that caveolae spatially control local Ca^2+^ release and vasodilation [124]. The activation of PKCε has been shown to mediate the internalization of vascular K_ATP_ channels (Kir6.1/SUR2B) via Cav-1, leading to channel inhibition. This process is disrupted by cholesterol depletion, dynamin mutations (K44E), or Cav-1 knockdown, further emphasizing the role of caveolae as essential platforms for channel trafficking [125].

### 3.4. Mechanosensitive Regulation of Ion Channels by Caveolae

Caveolae serve as mechanically deformable membrane invaginations, providing structural resilience to cells experiencing mechanical stress. These cholesterol-rich microdomains play a crucial role in mechanotransduction, buffering mechanical forces and modulating ion channel activity in response to changes in membrane tension. The loss or disruption of caveolae has been associated with increased mechanical sensitivity, dysregulated ion channel activity, and pathological remodeling in cardiovascular and muscular diseases.

Cav-3, the predominant scaffolding protein in muscle caveolae, plays a critical role in maintaining membrane stability and regulating mechanosensitive ion channels. Mutations in Cav-3 lead to limb-girdle muscular dystrophy, resulting in cytoskeletal defects that cause excessive activation of mechanosensitive cation channels and increased intracellular Ca^2+^ levels. While Cav-3 normally protects the sarcolemma from mechanical stress, its upregulation in dystrophic conditions may act as a compensatory mechanism to counteract mechanical instability [126]. In the cardiovascular system, caveolae regulate mechanotransduction in the sinoatrial node, integrating mechanical and biochemical signaling factors that influence cardiac automaticity. The presence of eNOS, NOX2-derived reactive oxygen species (ROS), and Ca^2+^ dynamics within caveolae is believed to suppress excessive mechanotransduction. However, the chronic loss of caveolae under sustained shear stress leads to uncontrolled activation of these signaling pathways, creating aberrant feedback loops associated with cardiac pathologies [127].

Caveolae function as a reserve to absorb mechanical forces and limit excessive activation of mechanosensitive channels. Disrupting caveolae, such as through cholesterol depletion, diminishes this reserve capacity, leading to accelerated cell swelling under hypotonic conditions and enhanced activation of swelling-activated chloride current [128]. Cav-3 has also been shown to influence SWELL1-mediated Cl^-^ currents, where its expression enhances SWELL1 activity, conferring resistance to swelling-induced membrane damage [71]. In cardiac myocytes, Cav-3 downregulation increases sensitivity, which may contribute to atrial fibrillation in hypertensive patients [72]. These findings highlight the importance of caveolae in maintaining cellular homeostasis under mechanical stress by modulating ion channel responsiveness.

Cav-1 serves as a scaffold for P2Y1 and P2Y2 receptors, stabilizing them in cholesterol-rich microdomains to regulate downstream signaling cascades involved in mucosal secretion, motility, and pain transmission. In alveolar cells, ATP release in response to mechanical expansion activates P2Y2 receptor-dependent Ca^2+^ signaling, which in turn regulates surfactant secretion, emphasizing the role of caveolae in lung mechanotransduction [129]. Piezo1, a key mechanosensitive channel implicated in cardiac hypertrophy and fibrosis, is similarly regulated by Cav-3. Under conditions of pressure overload or angiotensin II stimulation, Piezo1 relocates out of caveolae, leading to enhanced channel function and increased expression [73]. This suggests that caveolae act as regulatory compartments, restricting Piezo1 activity under normal conditions to prevent excessive mechanotransduction that contributes to pathological cardiac remodeling.

Caveolae also contribute to endothelial mechanosensitivity by regulating ENaCs. These channels reside in Cav-1-enriched microdomains, where their function is influenced by heme oxygenase activity. Under shear stress, HO-1 upregulation leads to increased carbon monoxide production, which enhances ENaC activity and promotes Na^+^ influx. Persistent ENaC activation is linked to endothelial dysfunction and vascular diseases, establishing a connection between caveolae disruption and pathological states such as atherosclerosis [39].

### 3.5. Caveolae-Mediated Regulation of Ion Channels by Other Channels and Proteins

Caveolae, specialized lipid microdomains in the plasma membrane, serve as platforms for the regulation of various ion channels. These lipid rafts not only facilitate channel trafficking and localization but also regulate their functional activity through interactions with other ion channels and membrane-associated proteins.

Caveolae facilitate cross-talk between different ion channels, influencing their activity and functional coordination. A notable example is the interaction between K_Ca_3.1 and BK_Ca_ channels, where K_Ca_3.1 activation suppresses BK_Ca_ channel activity. This cross-regulation is cholesterol-dependent and cytoskeleton-regulated, as cholesterol depletion weakens the interaction, whereas cholesterol enrichment enhances it, demonstrating that caveolae and lipid raft integrity are essential for inter-channel modulation [130]. In vascular smooth muscle cells, caveolae also facilitate the coupling between BK_Ca_ and LTCC, enabling coordinated regulation of vascular tone and excitation-contraction coupling. Cav-1 directly interacts with Ca_v_1.2 to regulate calcium signaling, forming a molecular complex that governs the spatial and temporal calcium dynamics necessary for smooth muscle excitability and contraction [131].

In endothelial cells, caveolae are integral to the regulation of ion channels that control vascular relaxation and mechanosensation. TRPV4 and K_Ca_2.3 channels, which are critical for endothelial-dependent vasodilation, co-localize within caveolae, forming a functional complex that contributes to coronary circulation regulation [61]. Additionally, ENaCs are mechanosensitive and reside in caveolae, where they modulate vascular function in response to shear stress. The upregulation of heme oxygenase-1 leads to increased carbon monoxide production, which enhances ENaC activity and sodium influx, potentially contributing to endothelial dysfunction and vascular disease [39].

Beyond direct ion channel interactions, caveolae also regulate channel trafficking and endocytosis through associated proteins. In cardiomyocytes, EHD2 interacts with caveolae to stabilize the localization of K_ATP_ channels, thereby influencing their trafficking and turnover. This regulatory mechanism highlights the broader role of caveolae in controlling ion channel distribution and function through protein interactions [64].

## 4. Caveolae and Diseases

### 4.1. Cardiovascular Diseases

**Figure 4:**
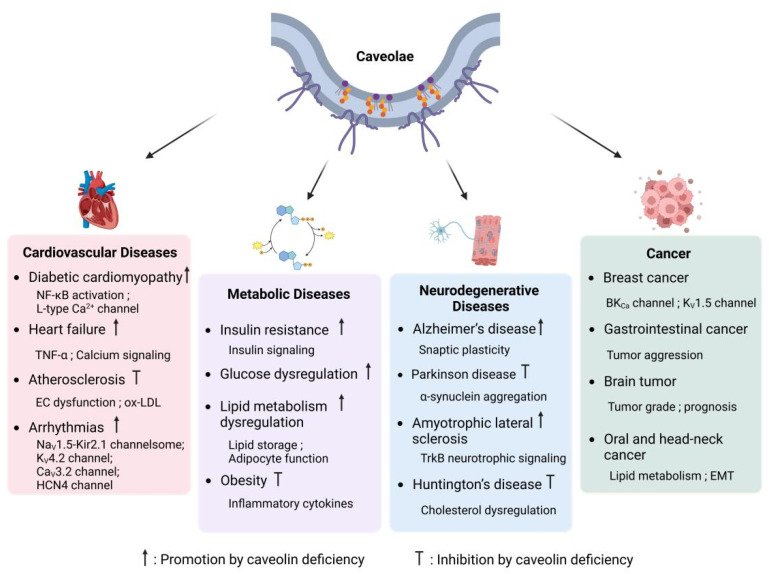
Caveolae-associated diseases. Caveolae are associated with various diseases, including cardiovascular diseases, metabolic disorders, neurodegenerative diseases and cancer, and play a regulatory role in their pathophysiology.

Caveolae are essential components in cardiovascular physiology, playing a pivotal role in regulating endothelial function, lipid metabolism, ion channel activity, and myocardial signaling. Dysfunction of caveolae is implicated in the pathogenesis of a wide range of cardiovascular diseases, such as atherosclerosis, diabetic cardiomyopathy, heart failure, and arrhythmias (Figure 4). By modulating mechanotransduction, inflammatory pathways, and the compartmentalization of ion channels, caveolae significantly influence disease progression and emerge as potential therapeutic targets for cardiovascular disorders.

#### 4.1.1. Atherosclerosis

Caveolae play a pivotal role in the initiation and progression of atherosclerosis, primarily through their involvement in lipid metabolism and endothelial dysfunction. It has been demonstrated that caveolae mediate the endocytosis and transcytosis of oxidized low-density lipoprotein (ox-LDL), promoting lipid accumulation within vascular cells [132]. Inhibition of this pathway, such as through Cav-1 silencing, significantly reduces ox-LDL uptake and foam cell formation, underscoring the contribution of caveolae to lipid-driven atherogenesis [133,134]. Beyond lipid transport, caveolae are critical regulators of vascular inflammation and remodeling. They participate in the activation of NF-κB signaling, a key pathway driving endothelial dysfunction and vascular inflammation [135,136]. Furthermore, caveolae modulate vascular smooth muscle cell (VSMC) behavior, where their dysfunction enhances VSMC proliferation and migration—processes closely linked to plaque instability and restenosis [135,137,138,139].

#### 4.1.2. Diabetic Cardiomyopathy

The role of caveolae in diabetic cardiomyopathy (DCM) is closely tied to their regulation of inflammatory signaling and myocardial remodeling. In diabetic mouse models, Cav-1 deficiency exacerbates cardiac dysfunction, characterized by increased hypertrophy, fibrosis, and inflammation [140]. This pathological progression is associated with enhanced NF-κB activation, which upregulates inflammatory and hypertrophic genes, further driving disease severity. Conversely, Cav-1 overexpression has been shown to mitigate these effects, highlighting its protective role in DCM. In cardiomyocytes, caveolae play a critical role in maintaining calcium homeostasis and excitation-contraction coupling. The disruption of caveolae, particularly through Cav-3 depletion, impairs T-tubule organization and L-type Ca^2+^ currents, resulting in calcium mishandling and exacerbating diabetic cardiomyopathy [141,142,143]. Furthermore, caveolae are implicated in the development of left ventricular hypertrophy and pulmonary hypertension. Their loss promotes excessive MAPK signaling and endothelial dysfunction, which collectively worsens cardiac remodeling [144].

#### 4.1.3. Heart Failure

Caveolae play a critical role in maintaining myocardial integrity, and their dysfunction is closely related to the progression of heart failure. In heart failure models, Cav-3 expression is significantly reduced, which correlates with deteriorating left ventricular function and T-tubule disorganization. In patients with heart failure, reduced levels of Cav-3 are associated with impaired calcium signaling and contractile dysfunction, underscoring its critical role in maintaining myocardial function. Moreover, overexpression of tumor necrosis factor-α (TNF-α) has been shown to contribute to Cav-3 depletion in failing hearts, suggesting an inflammation-mediated mechanism underlying caveolar loss. Given its essential functions in ion channel compartmentalization and T-tubule stabilization, Cav-3 deficiency leads to electrical instability and contractile impairment, further contributing to heart failure pathogenesis [145].

#### 4.1.4. Arrhythmias

Caveolae are essential for maintaining cardiac excitability, and their dysfunction is associated with the occurence of arrhythmias. Specifically, mutations or dysregulation of Cav-3, a key structural protein of caveolae, can significantly impair ion channel function, thereby inducing proarrhythmic alterations. For instance, Cav-3 mutations have been shown to diminish inward rectifier potassium currents [102,146], while enhancing late sodium currents, predisposing the heart to ventricular arrhythmias. Additionally, overexpression of Cav-3 can disrupt diastolic Ca^2+^ transients, leading to aberrant L-type Ca^2+^ channel activity and further exacerbating the propensity for ventricular arrhythmias [47,147,148]. Furthermore, Cav-3 deficiency disrupts HCN4 channel function, impairing pacemaker activity and contributing to conduction disorders [149,150]. Given the role of caveolae in ion channel compartmentalization, their disruption leads to electrical instability, increasing susceptibility to atrial and ventricular arrhythmias.

### 4.2. Metabolic Diseases

Caveolae play a pivotal role in the regulation of metabolic processes, serving as critical platforms for insulin signaling, lipid metabolism, and the modulation of inflammatory pathways. Dysfunction of caveolae has been strongly associated with the pathogenesis of metabolic disorders, including insulin resistance, obesity, and type 2 diabetes. These conditions are characterized by disrupted glucose homeostasis, aberrant lipid metabolism, and an elevated risk of cardiovascular complications. By orchestrating interactions between membrane-associated proteins and key signaling molecules, caveolae finely tune hormonal responses and energy balance, underscoring their essential role in preserving metabolic stability and overall physiological homeostasis.

#### 4.2.1. Insulin Resistance and Glucose Dysregulation

Caveolae play a critical role in the proper functioning of the insulin receptor (IR) and the facilitation of insulin-mediated glucose uptake. Specifically, the IR forms a dynamic complex with Cav-1 and ganglioside GM3 within caveolae, a structural arrangement essential for optimal receptor activity. Disruption of this tripartite interaction compromises IR mobility and stability, leading to impaired insulin signaling and subsequent metabolic dysregulation [151]. In insulin-resistant adipocytes, elevated levels of ganglioside GM3 disrupt the IR-Cav-1 complex, leading to reduced insulin sensitivity and contributing to adipose tissue insulin resistance, a hallmark of metabolic syndrome. Similarly, in the heart, insulin signaling is critically dependent on caveolae. Under high-fat diet conditions, Cav-3 dissociates from the insulin receptor β-subunit, thereby impairing insulin-mediated cardioprotective effects. This pathological dissociation is associated with tyrosine nitration at Tyr73 in Cav-3, a modification that disrupts IR complex formation and inhibits downstream insulin signaling pathways [152]. Restoration of Cav-3 expression or inhibition of its nitration rescues insulin signaling and provides protection against diabetes-induced ischemic heart failure, underscoring the importance of maintaining caveolae integrity for preserving insulin sensitivity in cardiac tissues. Additionally, genetic variations in Cav-1 have been implicated in systemic insulin resistance. Epidemiological studies in hypertensive populations have demonstrated that Cav-1 single nucleotide polymorphisms (SNPs) are associated with elevated fasting insulin levels, increased homeostasis model assessment of insulin resistance scores, and impaired glucose disposal during insulin clamp studies [153]. In Cav-1 knockout mice, metabolic dysfunctions are intensified, indicating that genetic variations in Cav-1 may act as biomarkers for the early identification and management of insulin resistance and metabolic disorders. In skeletal muscle, the predominant isoform is Cav-3, and its deficiency causes considerable impairment in glucose uptake, which in turn leads to diminished insulin-stimulated signaling, glucose intolerance, and dyslipidemia [154]. These results emphasize the importance of caveolae in preserving insulin sensitivity and regulating glucose metabolism in muscle tissue, underscoring Cav-3 as a crucial element in muscle insulin resistance and overall metabolic health.

#### 4.2.2. Obesity and Lipid Metabolism Dysregulation

Caveolae are essential for lipid storage and adipocyte function, with Cav-1 and Cav-3 being vital for maintaining adipose tissue balance. In cases of obesity, there is a marked increase in Cav-1 expression, which is associated with elevated levels of pro-inflammatory cytokines like TNF-α and the activation of NF-κB [155]. Inflammatory pathways contribute to the dysfunction of adipose tissue and the development of metabolic syndrome, reinforcing the importance of caveolae in regulating adipose inflammation and lipid metabolism. In contrast, the absence of Cav-1 offers protection against obesity caused by diet. Investigation involving Cav-1 knockout mice demonstrates a lean phenotype, resistance to obesity from high-fat diets, and notable decreases in adipose tissue mass [31]. These mice exhibit severe lipid metabolism defects, including reduced triglyceride storage, elevated circulating free fatty acids, and impaired lipid droplet formation. Mechanistically, Cav-1 loss disrupts adipocyte caveolae, impairing lipid uptake and storage, which may contribute to altered systemic lipid homeostasis.

In pediatric populations, Cav-1 gene polymorphisms have been associated with metabolic syndrome in obese children [156]. This suggests that caveolae dysfunction may contribute to early-onset obesity and metabolic disturbances, reinforcing their role as potential therapeutic targets for metabolic disease prevention.

### 4.3. Neurodegenerative Diseases

Caveolae, as cholesterol-rich membrane microdomains, play a crucial role in neuronal signaling, synaptic stability, and neuroprotection. The structural protein Cav-1 regulates various signaling pathways involved in synaptic plasticity, neurotransmitter receptor function, and neuronal survival. Dysregulation of caveolae is implicated in the progression of multiple neurodegenerative diseases, including Alzheimer’s disease (AD), Parkinson disease (PD), amyotrophic lateral sclerosis (ALS), and Huntington’s disease (HD).

#### 4.3.1. Alzheimer’s Disease and Cognitive Decline

Cav-1 plays a crucial role in regulating synaptic plasticity and signaling for neuronal survival. It organizes glutamate receptors, neurotrophic factor receptors, and pre-survival kinases, which enhances cAMP production and promotes dendritic development [157]. Elevated levels of Cav-1 in neurons boost synaptic signaling and plasticity, resulting in improved memory and cognitive abilities in Alzheimer’s disease models. In contrast, the absence of Cav-1 results in accelerated neuronal aging, heightened accumulation of Aβ and phosphorylated Tau, and a decrease in hippocampal synapses, resembling the pathology associated with Alzheimer’s disease [158].

In aging brains, Cav-1 deficiency disrupts membrane lipid raft organization, impairing the localization of PSD-95, NMDA receptor (NR2A/NR2B), and BDNF receptors (TrkBR), which are critical for synaptic function [158]. Targeted re-expression of Cav-1 in Cav-1 null neurons restores synaptic integrity and reduces Aβ accumulation, supporting its role as a potential neuroprotective target in AD.

Cognitive decline in aging is linked to MLR disorganization, synaptic loss, and impaired long-term potentiation [159]. Cav-1 overexpression in aged mice improves neuroplasticity and enhances learning and memory associated with the hippocampus, whereas its deficiency leads to accelerated cognitive decline. These results indicate that Cav-1 plays a vital role in maintaining cognitive resilience and may serve as a promising therapeutic target for neurodegenerative conditions related to aging.

#### 4.3.2. Parkinson Disease and α-Synuclein Aggregation

Parkinson disease (PD) is characterized by the progressive accumulation and intercellular transmission of misfolded α-synuclein, a hallmark of its pathogenesis. Cav-1 expression increases with age and is upregulated in α-synuclein-overexpressing PD models [160]. Cav-1 overexpression promotes neuronal α-synuclein uptake and the formation of Lewy body-like inclusions, underscoring its significance in the propagation of α-synuclein and the advancement of the disease.

Mechanistically, Cav-1 phosphorylation at Y14 is essential for α-synuclein uptake and aggregation, suggesting a direct involvement of caveolae in PD pathology. These findings provide new insights into the molecular mechanisms of α-synuclein intercellular transmission, implicating Cav-1 as a potential modulator of PD progression.

#### 4.3.3. Amyotrophic Lateral Sclerosis (ALS) and Neuroprotection

In ALS, Cav-1 exerts neuroprotective effects by preserving neuromuscular function. Overexpression of Cav-1 in ALS mouse models delays disease progression, improves motor function, and enhances survival [161]. Cav-1 upregulation maintains muscle innervation, sustains TrkB neurotrophic signaling, and protects mitochondrial function, demonstrating its essential role in preventing ALS-related neuronal degeneration. These findings indicate that Cav-1 gene therapy may offer a novel therapeutic strategy for ALS, by maintaining neurotrophic signaling and stabilizing synaptic function.

#### 4.3.4. Huntington’s Disease and Cholesterol Dysregulation

Huntington’s disease (HD) is characterized by abnormal cholesterol metabolism and vesicular trafficking deficits. Mutant huntingtin directly interacts with Cav-1, leading to intracellular cholesterol accumulation and impaired caveolae-dependent endocytosis [162]. This interaction disrupts lipid homeostasis and neuronal function, contributing to HD progression. In HD models, Cav-1 loss or reduction rescues neuronal cholesterol abnormalities and significantly delays motor dysfunction and intracellular inclusion formation [163]. These findings suggest that targeting Cav-1-mediated cholesterol regulation could provide a therapeutic avenue for HD.

### 4.4. Cancer

Caveolae have a complex role in cancer progression, affecting tumor growth, invasion, metastasis, and treatment resistance. involvement varies depending on the tumor type, stage, and microenvironment, functioning as either tumor suppressors or promoters. In certain cancers, reduced caveolae levels are associated with heightened proliferation and angiogenesis, while in others, increased levels contribute to improved survival and metastatic capabilities [164].

In breast cancer, caveolae have been implicated in tumor progression and metastatic potential. Recent findings indicate that Cav-1 acts as a critical regulator of late-stage breast cancer metastasis, with its phosphorylation state playing a pivotal role. Specifically, potassium channel activation-dependent Cav-1 dephosphorylation was observed in highly metastatic breast cancer cells, suggesting that targeting this pathway could be a novel therapeutic approach to inhibit cancer cell migration and enhance contact inhibition [165]. Moreover, siRNA-mediated silencing of Cav-1 results in the activation of BK_Ca_ channels and enhances their surface expression, thereby driving breast cancer cell proliferation and invasiveness. In contrast, upregulation of Cav-1 suppresses BK_Ca_ channel activity and reduces their surface localization, ultimately inhibiting cancer progression [166]. Furthermore, in MCF-7 breast cancer cells, the co-expression of Cav-1 and K_v_1.5 significantly enhances cell survival. In contrast, overexpression of K_v_1.5 alone does not markedly influence cell viability, highlighting a potential regulatory role of Cav-1 in ion channel-mediated mechanisms that promote cancer cell survival [167].

In brain tumors, their expression has been associated with tumor grade and prognosis. In glioblastomas, they contribute to tumor progression, therapy resistance, and blood-tumor barrier regulation, making them a factor in treatment challenges [168]. Their presence in lower-grade astrocytomas and oligodendrogliomas is inconsistent, with some indications pointing to a possible tumor-suppressive role in the initial stages. Conversely, expression levels rise in high-grade tumors, which is associated with more aggressive characteristics. In meningiomas, elevated expression levels have been associated with a higher risk of recurrence and poorer survival rates.

In gastrointestinal cancers, including esophageal and pancreatic cancer, as well as hepatocellular carcinoma, elevated expression levels correlate with poor prognosis and increased tumor aggression. Conversely, in colorectal cancer, stromal expression seems to play a protective role, possibly affecting interactions within the tumor microenvironment [169]. This variability underscores the intricate nature of their functions across various tumor types. Their involvement in soft-tissue sarcomas and mesenchymal tumors suggests a role in cellular homeostasis, as they are highly expressed in benign mesenchymal tumors such as fibromas and lipomas [170]. However, in aggressive sarcomas, their role is more complex, with studies indicating both tumor-suppressive and tumor-promoting effects depending on the cellular and molecular context. In oral and head-neck cancers, caveolae-related mechanisms are implicated in lipid metabolism and epithelial-mesenchymal transition (EMT), particularly in early tumor development [171]. Their regulation of cell adhesion and migration further supports their role in metastasis and invasion. A significant aspect of their function in cancer is their response to hypoxia and therapy resistance. Under hypoxic conditions, they regulate membrane protein internalization and influence cellular adaptation to stress, independent of hypoxia-inducible factor signaling [172]. This contributes to tumor cell survival, drug resistance, and metastatic potential. Additionally, their involvement in the unfolded protein response and endoplasmic reticulum stress pathways has been linked to tumor cell survival under adverse conditions, further supporting their role in therapy resistance [173].

## 5. Conclusions and Future Perspectives

Caveolae serve as versatile regulatory platforms that influence ion channel activity through multiple mechanisms. Caveolins offer a structural framework to engage directly with ion channels, affecting their gating and transport. Additionally, caveolae establish a distinct lipid microenvironment that adjusts channel function in reaction to mechanical and biochemical signals. This multi-tiered regulation allows for accurate management of electrical signaling, mechanotransduction, and metabolic equilibrium. Future research should aim to delineate the intricate molecular structure and dynamic formation of caveolae-associated complexes through cutting-edge imaging and proteomic techniques, while also examining how these processes differ among various tissues and disease conditions. These findings are anticipated to reveal new therapeutic targets for reinstating ion channel functionality in a range of disorders.

## Data Availability

No new data were created or analyzed in this study.
